# Targeted Engagement of the Action Selection Network during Task-Oriented Arm Training after Stroke

**DOI:** 10.1155/2020/8814158

**Published:** 2020-09-23

**Authors:** Jill Campbell Stewart, Kaci Handlery, Jessica F. Baird, Erika L. Blanck, Geetanjali Pathak, Stacy L. Fritz

**Affiliations:** ^1^Physical Therapy Program, Department of Exercise Science, University of South Carolina, 921 Assembly Street, Room 301D, SC, 29208 Columbia, USA; ^2^Department of Exercise Science, University of South Carolina, Columbia, SC 29208, USA; ^3^Department of Cell Biology and Anatomy, School of Medicine, University of South Carolina, Columbia, SC 29208, USA

## Abstract

Action selection (AS), or selection of an action from a set of alternatives, is an important movement preparation process that engages a frontal-parietal network. The addition of AS demands to arm training after stroke could be used to engage this motor planning process and the neural network that supports it. The purpose of this case series is to describe the feasibility and outcomes associated with task-oriented arm training aimed at engaging the AS behavioral process and the related neural network in three individuals with chronic stroke. Three participants with mild to moderate motor deficits completed 13 to 15 sessions of task-oriented arm training that included AS cues for each movement repetition; cues dictated movement direction, height, or distance. Before and after training, individuals completed an AS brain-behavior probe during functional MRI. AS behavioral performance improved after training (increased accuracy, decreased reaction time) in all participants while brain activation in the AS network (dorsal premotor, parietal, dorsolateral prefrontal cortices) decreased in two participants. Gains in motor function were also found in all three participants, especially on patient-reported measures of perceived difficulty and confidence to complete upper extremity functional tasks. It was feasible to target the AS behavioral process and the related neural network through the addition of AS demands to functional, task-oriented arm training in three individuals with mild to moderate motor dysfunction poststroke.

## 1. Introduction

Successful performance of skilled motor tasks involves a variety of movement preparation processes in addition to movement execution. Action selection (AS), or selection of an action from a set of alternatives, is one of these movement preparation processes [1]. The selection of the action to be performed (e.g., to reach and pick up a cup of water) must occur for the movement parameters to be specified (e.g., descending motor commands to the arm and hand). While some degree of action selection is required for all skilled actions, one can relatively increase AS demands by requiring that a movement response be selected based on an external, abstract visual rule (e.g., selecting to pick up the cup of water from either the low shelf or the high shelf based on an external cue). Task conditions that increase AS demands lead to a longer movement preparation phase and have consistently been shown to engage a network of frontal and parietal brain regions with dorsal premotor cortex (PMd) thought to be a key node in this network [2–5]. Older adults and individuals post stroke engage a similar network during the performance of an AS task [6], however, some individuals also activate dorsolateral prefrontal cortex (DLPFC), suggesting engagement of cognitive resources during AS. Therefore, a practice condition that includes AS demands during training has the potential to engage and alter premotor and prefrontal activation.

Task-oriented training, the practice of goal-directed, functional tasks, is an important component of arm and hand rehabilitation after stroke [7–9]. While the amount of training (number of movement repetitions) may be a key factor in determing the effectiveness of task-oriented arm training [10–13], the optimal content of training remains unknown. Changes in task conditions or practice instructions can alter behavioral performance of goal-directed tasks [14–18] and modulate neural activation [19–22] in individuals poststroke, however, most research in this area has been cross-sectional in nature or utilized laboratory tasks that may not translate into clinical practice. In nondisabled individuals, practice conditions that engage greater neural resources during practice may lead to better behavioral performance and reduced brain activation on retention [23–25], however, such approaches to functional arm training after stroke have not been systematically investigated. The addition of AS demands to task-oriented arm training may provide an avenue to increase the challenge level of training by requiring additional movement planning and increasing engagement of the brain regions that support AS (premotor, parietal, and prefrontal cortices).

The addition of AS cues to task-oriented arm training may provide the opportunity to target the AS behavioral process and the brain regions that support this process within the context of functional movement training aimed at improving motor function. We chose to add AS demands to training as this movement preparation process is important in the performance of goal-direction actions [1], provides a clear, well-defined variation in practice condition, and has a robust literature on its neural correlates [2–5, 26, 27], including PMd, a brain region thought to contribute to motor function after stroke [28–31]. The purpose of this case series is to describe the feasibility and outcomes associated with task-oriented arm training aimed at engaging the AS behavioral process and AS neural network through the addition of AS cues to each movement repetition in three individuals with arm motor deficits due to stroke.

## 2. Methods

### 2.1. Participants

Three right-hand dominant individuals with chronic left hemisphere stroke were recruited from the local community. All three participants completed an AS brain-behavior probe to examine AS behavioral performance and brain activation with the contralesional right hand before and after a 3-week training phase. Clinical assessments of arm motor function were also completed before (two baseline assessments 1 week apart) and after training as well at a 3-week follow-up. Participants provided written informed consent according to a study protocol approved by the University of South Carolina Institutional Review Board (Protocol ID# Pro00032516).

Individuals were eligible to participate if they were ≥18 years old, had a stroke at least 6 months prior to enrollment, were right-hand dominant [32] prior to stroke, showed evidence of upper extremity impairment as defined by an Upper Extremity Fugl-Meyer (UEFM) Motor Score < 66 [33] and/or at least a 15% deficit on the Nine Hole Peg Test [34] on the more impaired hand compared to the less impaired hand, and demonstrated some movement ability as shown by an UEFM score > 30 and/or the ability to move at least one block on the Box & Blocks Test [35]. Individuals were excluded if they had any acute medical problems, severe ideomotor apraxia as defined by a score ≤ 65 on the Test of Upper Limb Apraxia [36], hemispatial neglect with a score < 52 on the Behavioral Inattention Test Star Cancellation [37], significant arm pain that interfered with movement, contraindications to MRI scanning (e.g., metal implants or claustrophobia), or a history of other non-stroke related neurological disorders.

A summary of initial presentation for the three participants is presented in Table 1. S1 (54-year-old Caucasian male) presented with mild motor impairment (based on UEFM Motor Score [38]), deficits in right arm function, sensory deficits, and no apraxia as measured by the Test for Upper Limb Apraxia (see Supplemental Figure 1 for lesion location). S1 reported difficulty in using his hand to perform functional tasks and reduced confidence to complete functional tasks that require the arm and hand. S2 (67-year-old Caucasian male) presented with mild motor impairment and relatively mild deficits in arm function; he did not have sensory deficits or apraxia. S2 reported continued difficulty in using the hand to perform functional tasks and moderate to good confidence in his ability to complete functional tasks that require the arm and hand. S3 (63-year-old African American female) presented with mild to moderate motor impairment and decreased arm function; she did not have sensory deficits or apraxia. S3 reported that using the weaker hand to perform functional tasks was somewhat difficult and reported moderate to good confidence in her ability to complete functional tasks with the arm and hand.

### 2.2. Task-Oriented Training with Action Selection Demands

The intervention involved 15 1.5-hour sessions of task-oriented training (5 times per week for 3 weeks) that focused on arm and hand function with a licensed physical therapist. Task-oriented arm training involves the repetitive practice of goal-directed, functional movements [7, 8]. Any functional task that required movement of the weaker upper extremity could be included; both unimanual and bimanual tasks were part of training. Tasks focused on proximal arm control (i.e., shoulder/elbow movement), hand grasp (gross grasp, fine motor), object manipulation (tool use, movement of objects within the hand), or the combination of proximal control and hand grasp/object manipulation [39]. Training was designed to be individualized, challenging, and progressive. Tasks completed during training sessions were chosen based on the level of motor ability and participant preferences and goals. The difficulty level of the motor training was progressed across sessions through changes in task set-up (e.g., moving from reaching at midline to reaching across midline), task demands (e.g., increase weight of object lifted), and the task itself (e.g., reach and pick up a cup with a gross grasp to reach and pick up a coin with a pincer grasp while holding additional coins in the same hand) [39]. Participants completed 4 to 5 tasks per session, and all training was completed in 10 trial blocks. Rest was provided between tasks or when requested by the participant.

**Figure 1:**
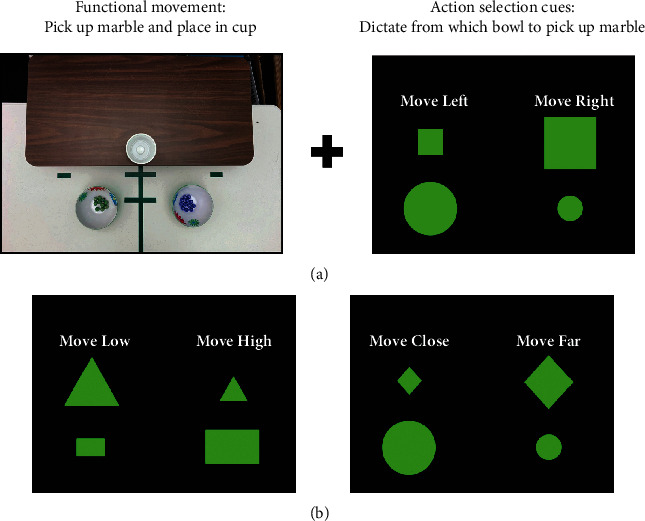
Example of training task with action selection cues. (a) Training involved task-oriented training (e.g., functional movement of picking up a marble and placing it into a cup) with the addition of action selection demands to each movement repetition (e.g., visual cues dictated whether the marble should be picked up from the right bowl or the left bowl on each movement repetition). (b) The action selection rule varied (shapes and rules were changed to maintain challenge) and was used to provide selection of movements to the right or left, to a low surface or a high surface (e.g., pick up marble from the low table or the high table), or to a close location or a far location (e.g., pick up a marble from a close bowl or a far bowl).

**Figure 2:**
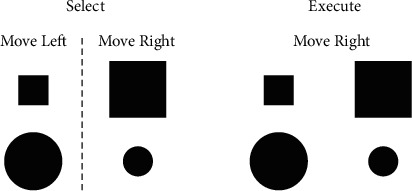
Select and Execute cues used during the brain-behavior probe. During Select, movement direction was dictated by an abstract rule (large square or small circle = move right; small square or large circle = move left). During Execute, movement direction was the same on every trial regardless of visual cue. The same Select cue set was used in the first arm training session.

Action selection cues were added to every movement repetition during training. Cues were displayed on a screen directly in front of the participant using E-Prime 2.0.10 (Psychology Software Tools, Sharpsburg, PA) and were designed to require movement selection between right and left, close and far, or high and low (Figure 1; Table 2). For example, the cue presented on the screen would direct the participant to pick up the cup on the right instead of the cup on the left. Therefore, the individual had to attend to the cue, remember the rule presented (which cue indicated right and which cue indicated left), and select a response for every movement repetition. In an effort to maintain the engagement of the AS network throughout training, an algorithm was used to progress cue difficulty (Supplemental Table 1). On the first day of training, all participants began with the same cue mapping used in the brain-behavior probe (Level 5: small square-large circle/large square-small circle; Figure 2). If the participant was more than 85% accurate with this cue mapping, a new set of cues/shapes was introduced on the next training day (Level 6: triangles/squares, diamonds/crosses, etc.); if the participant was less than 85% accurate, he or she continued with the same cue mapping until this accuracy level was achieved. Once participants were more than 95% accurate with the new cue set, a novel cue set was provided for each task practiced during a single training session (Level 7). Finally, once participants were more than 95% accurate at this level, a new cue set was provided for each 10-trial block of practice during a training session (Level 8). If a participant had difficulty with the 4-cue mapping on Day 1 of training (<70% accurate for movement selection), the difficulty of the AS cues was decreased to a 2-cue mapping on Day 2 (Level 1); none of the three individuals in this case series required this step.

### 2.3. Action Selection Brain-Behavior Probe

Before and after the motor training period, participants completed an AS brain-behavior probe with the more impaired hand to examine AS behavioral performance and brain activation during AS. The behavioral task involved right or left movement of a joystick based on a visual cue in two different conditions. In the Select condition, the individual moved right or left based on an abstract rule (Figure 2). When a small square or large circle was shown, a joystick movement was made to the left; when a large square or small circle was shown, a joystick movement was made to the right. In the Execute condition, the visual cues were the same, however, the individual made a joystick movement in the same direction on every trial irrespective of the size/shape of the cue (no rule to follow, minimal AS demands). In both conditions, a single cue was presented for 2 sec; the intertrial interval varied between 2.0 and 3.5 sec to minimize anticipatory responses prior to the cue. Data from joystick movements were analyzed using a custom script in Matlab (MathWorks, Natick, MA; see Supplemental Methods). Response accuracy (correct movement direction), reaction time (RT; movement onset time-cue onset time), and RT cost (Select RT-Execute RT; representing the relative increase in RT from the Execute condition to the Select condition for each individual) were compared from pretraining (Pre-TX) to posttraining (Post-TX). Prior to the intervention phase, participants practiced the AS behavioral task for four consecutive days (3 blocks of 24 trials in each condition on Day 1; 5 blocks of 24 trials in each condition on Days 2 to 4) for a separate research question on short-term practice [40]; only Day 1 data (Pre-TX) is presented in this case series as it best represents initial performance on the AS behavioral probe task.

Before (Pre-TX) and after (Post-TX) the period of motor training, individuals completed the action selection behavioral task in a 3T Siemens Prisma MRI scanner with a 20-channel head coil and an MRI compatible joystick. Data from the MRI joystick was not available due to technical issues; therefore, task performance was quantified using data collected just prior to the MRI session. In a previous study using the same task in individuals post-stroke, the relative performance between the Select and Execute conditions was similar during functional MRI and practice in the lab just prior [6]. Functional MRI images were acquired in a block design (TR = 1,000 ms, TE = 37 ms, 56 slices, acquisition voxel size 2.8 × 2.8 × 2.5 mm); 24 sec of movement (green cues) alternated with 24 sec of rest (red cues) separated by periods of fixation (white cross for 8 sec). Each individual completed four functional MRI runs, two in the Select condition and two in the Execute condition in alternating order; S2 and S3 completed Execute in the first run while S1 completed Select in the first run. Structural brain images were also acquired and included T1 (TR = 2250 ms, TE = 4.11 ms, 192 slices, 1 mm^3^ isotropic voxels) and T2 (T2 = 3200 ms, TE = 567 ms, 176 slices, 1 mm^3^ isotropic voxels) structural scans for lesion identification and normalization of functional images. Diffusion weighted images (TR = 3839 ms, TE = 71 ms, 68 slices, 1.8 mm^3^ isotropic voxels, 56 noncollinear directions, *b* = 1000 s/mm^2^) were acquired and used for the determination of corticospinal tract integrity.

All functional imaging data were analyzed using SPM12 (Wellcome Trust Centre for Neuroimaging, London, UK). First, the origin of the structural T1 image was checked and repositioned to the anterior commissure as needed. Volumes from each fMRI run were realigned and resliced to the first volume to account for motion artifact. Next, the realigned and resliced images were coregistered to the participant's structural T1-weighted image. The participant's structural image was normalized to a T1 older brain template with the stroke lesion masked out using the Clinical Toolbox in SPM [41]. The normalization parameters were then applied to all the realigned, resliced, and normalized functional volumes for each run. The normalized images were resampled to 2 mm^3^ voxels. Images were then spatially smoothed with an isotropic Gaussian filter (FWHM 8 mm), and a temporal filter was applied (1/128 Hz) to remove low-frequency confounds. Data from each functional run were inspected for outliers due to excessive head motion (>2 mm translation or >0.2 radian rotation between volumes) or signal noise (*Z* > 5 from the mean image intensity) using the Artifact Detection Tool toolbox (http://www.nitrc.org/projects/artifact_detect); outliers were deweighted during statistical analysis.

First-level statistical analysis was performed separately for each participant using a general linear model [42, 43]. For each run, movement and rest epochs were modeled separately against fixation for later contrast. To determine the regions active during each condition (Execute, Select), movement was contrasted with rest (Move > Rest); both runs for each condition were weighted equally in all contrasts. The first derivative of head motion for all six directions was added as a regressor of no interest to account for the effect of head motion in the data. To examine changes in activation in the action selection network during movement after training, a region-of-interest analysis was used. The peak of activation in each region (bilateral PMd, bilateral DLPFC, and bilateral parietal) during Select before training was selected for each individual (Supplemental Table 2); no region-of-interest used in analyses was lesioned. Activation during movement was extracted as percent signal change (6 mm radius sphere centered on the peak) during each condition (Execute, Select) on each day (Pre-TX, Post-TX) using the Marsbar toolbox [44].

### 2.4. Outcome Measures

The feasibility of task-oriented training that includes AS demands was examined through the assessment of the Select cue progression, Select accuracy, and total movement repetitions per session. Changes in AS task performance were assessed by performance on the AS brain-behavior probe while changes in the neural resources required to complete the AS task were assessed by the magnitude of brain activation in bilateral PMd, DLPFC, and parietal cortices.

Changes in clinical measures of arm and hand function were also assessed and compared to published values for minimally clinically important difference (MCID), an indication that the change was meaningful, or minimal detectable change (MDC), an indication that the change was greater than measurement noise, for each measure if available. The three primary outcome measures, Action Research Arm Test (ARAT), Box & Blocks Test (BBT), and the Stroke Impact Scale (SIS) Hand Domain, were measured twice at baseline to establish stability of these measures prior to training, after training, and at a 3-week follow-up. The ARAT [45] and the BBT [35] are measures of upper extremity function with an MCID of 5.7 [46] and an MDC of 6 [47], respectively. The SIS Hand Domain [48] is a patient-reported outcome measure that indicates perceived difficulty in completing functional tasks with the more impaired hand with scores that range from 0 (could not do) to 100 (not difficult); the MCID for the SIS Hand Domain is 17.9 [49]. Secondary measures included the UEFM Motor Score [33] to assess changes in motor impairment (MCID = 5.25 [50]), the Nine-Hole Peg Test [34] to assess finger dexterity, and the Confidence in Arm and Hand Movements (CAHM) to assess patient-reported confidence in the ability to complete functional arm tasks (MCID = 7.3 [51]). The CAHM is a 20-item questionnaire that asks the individual to rate his or her level of confidence to perform a series of functional tasks that involve the weaker arm or both arms on a scale of 0 to 100 (0 indicates “very uncertain/unconfident” while 100 indicates “very certain/confident” about being able to successfully perform a task).

## 3. Results

### 3.1. Feasibility of Motor Training That Includes Action Selection Demands

**Figure 3:**
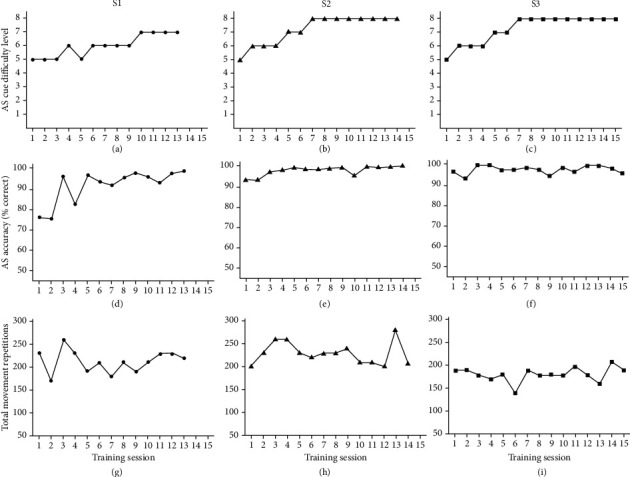
Training data by session and participant including action selection (AS) cue difficulty level (a–c), AS accuracy (d–f), and total number of movement repetitions (g–i).

All three participants tolerated task-oriented arm training that included AS demands. Individuals completed at least 13 training sessions (S1 = 13, missed two sessions due to conflict with other appointments; S2 = 14, missed one session due to illness; and S3 = 15, completed all sessions). AS cue difficulty was progressed over sessions, individualized for each participants' accuracy during training (Figure 3). S2 and S3 both progressed to the most difficult AS cue level (Level 8: new cue set every 10-trial block) with cue accuracy (correct movement selection) above 90% across sessions. S1 had lower accuracy, especially in the early training sessions, but progressed in AS cue difficulty over sessions achieving Level 7 (new cue set every task). The number of movement repetitions was maintained across sessions, even as AS cue difficulty was progressed (Figure 3). Mean repetitions per session was the lowest for S3 (181.3 ± 16.4) who presented with the greatest level of motor impairment compared to S1 (212.3 ± 24.9) and S2 (229.3 ± 24.0).

### 3.2. Action Selection Performance Changes in Response to Motor Training with Action Selection Demands

**Figure 4:**
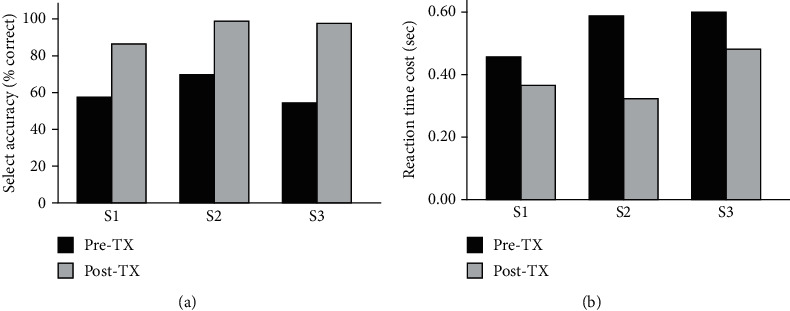
Select performance on the action selection brain-behavioral probe task before (Pre-TX) and after (Post-TX) training. Select accuracy (a) represents responses in the correct direction (right/left); reaction time cost (b) represents the increase in reaction time to complete the Select condition compared to the Execute condition (Select RT-Execute RT).

All three participants improved AS behavioral performance on the AS probe task from Pre-TX to Post-TX. Prior to training, as expected, accuracy was lower and RT was longer for the Select condition compared to the Execute condition (Supplemental Figure 2). All three participants showed an increase in accuracy for the Select condition after training (Figure 4). Starting with the lowest RT cost at Pre-TX (0.455 sec), S1 had a 90 msec decrease in RT cost (Select RT-Execute RT) from Pre-TX to Post-TX (Figure 4). S2 and S3 had similar RT cost at Pre-TX (~590 msec) with S2 showing a greater decrease (269 msec decrease) in cost after training than S3 (114 msec decrease).

**Figure 5:**
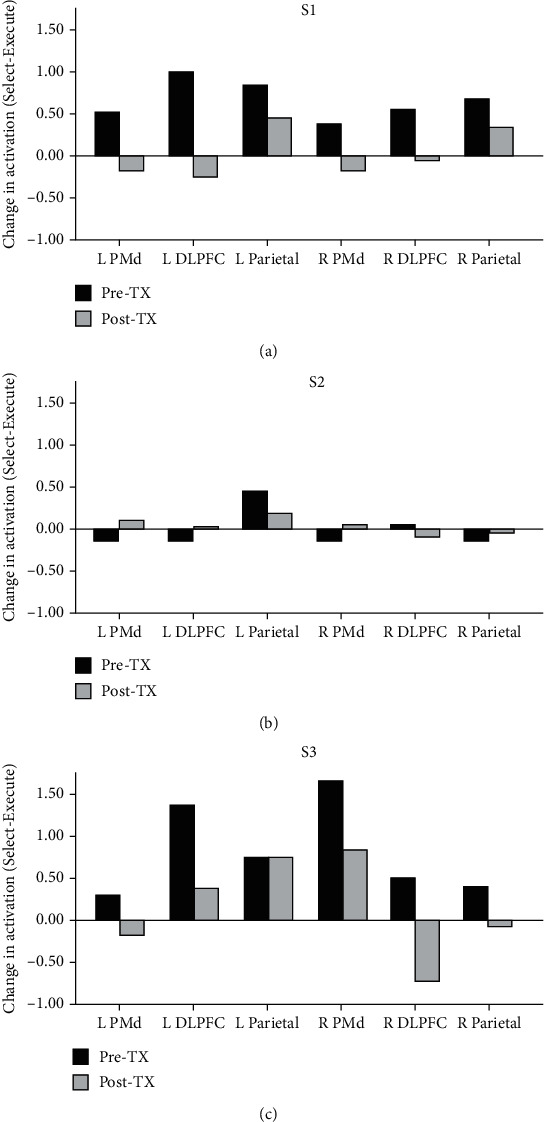
Brain activation in regions of interest during Select performance with the more impaired hand before (Pre-TX) and after (Post-TX) training for S1 (a), S2 (b), and S3 (c). Each bar represents the change in activation during the Select condition relative to the Execute condition on each day. Left (L) is the ipsilesional side. Right (R) is the contralesional side. PMd = dorsal premotor cortex; DLFPC = dorsolateral prefrontal cortex.

Before training, premotor, prefrontal, and parietal activation tended to increase bilaterally during completion of the Select condition compared to the Execute condition for S1 and S3 but remained relatively unchanged for S2 (Figure 5; Supplemental Figure 3). After training, the increase in activation from the Execute to the Select condition was less in premotor, prefrontal, and parietal brain regions for S1 and S3. S2 had the lowest level of brain activation overall and showed minimal change in activation when moving from the Execute to the Select condition before training; the activation pattern for this individual did not change with training.

### 3.3. Motor Function Changes in Response to Motor Training with Action Selection Demands

S1 did not show changes on the ARAT or the BBT but did show a decrease in the time to complete the Nine-Hole Peg (7 sec), a measure of finger dexterity, after training (Table 3). S1 also showed improvements in patient-reported measures of perceived difficulty to complete tasks with the more impaired hand (SIS Hand Domain increase of 20 points) and increased confidence to complete upper extremity based functional tasks (CAHM increase of 20 points). These changes were greater than the reported MCID values and were maintained at follow-up. S2 had small improvements in motor impairment (UEFM increase of 4 points) and motor function (BBT increase of 5 blocks) after training, but these changes did not exceed the MCID values. This individual did show an improvement in finger dexterity post-treatment (Nine-Hole Peg time decrease of 13.4 sec) but not at follow-up. S2 showed improvements in perceived difficulty to complete tasks with the more impaired hand (SIS Hand Domain increase of 20 points), and this improvement was maintained at follow-up. S3 had gains in motor function as measured with the ARAT after training (increase of 7 points) that exceeded the MCID and was maintained at follow-up. Improvements in finger dexterity as measured by the Nine-Hole Peg were also seen at post-treatment (decrease of 4.5 sec) and follow-up (decrease of 10.3 sec) assessments. S3 reported a decrease in the difficulty to complete tasks with the more impaired hand (SIS Hand Domain increase of 35 points) and an increase in confidence to complete functional tasks with the upper extremities (CAHM increase of 22 points). These improvements in patient-reported outcome measures were greater than the MCID and maintained at follow-up.

## 4. Discussion

Overall, it was feasible to add AS demands to task-oriented arm training in three individuals with mild to moderate motor impairment due to stroke. After training, AS behavioral performance improved (increased accuracy, decreased RT cost), and the neural resources utilized during AS decreased in bilateral premotor, prefrontal, and parietal cortices, suggesting the training paradigm was successful in targeting the AS behavioral process and its related neural network during training. Gains in motor function were also found in all three participants, especially in patient-reported measures of perceived difficulty and confidence, suggesting that motor function improvements were possible in the presence of the added action selection demands during training.

All three participants showed improvement on the AS brain-behavior probe after training. Behavioral performance on the joystick-based AS task improved from pre- to posttraining (increased accuracy, decreased RT cost) in all three participants while brain activation in key regions (premotor, prefrontal, and parietal) decreased in two participants. Overall, these findings suggest that these individuals were able to perform the motor preparation process of AS more accurately and more quickly, utilizing less neural resource, after training. Overactivation during movement has frequently been reported in individuals post-stroke, both during simple movement execution tasks and in response to an increase in task demands [21, 52, 53]. In nondisabled individuals, practice conditions that engage greater neural resources during practice may lead to better behavioral performance and reduced brain activation on retention [23–25]. Training paradigms that add demands to movement that increase brain activation during training after stroke, such as through the addition of AS demands, may benefit behavioral performance and/or reduce the neural resources required for movement after training.

Gains in motor function were found despite the addition of AS demands to task-oriented training. Improvements in patient-reported outcome measures of perceived difficulty to complete hand tasks (SIS Hand domain) and confidence to complete upper extremity functional tasks (CAHM) were seen in all three individuals after training. Improvements in motor function were more variable, but all three participants improved performance on at least one measure (Nine-hole peg, ARAT). Patient-reported outcome measures may be more sensitive to deficits than performance outcome measures in individuals with mild motor impairment [54], although it is possible that changes in these self-report measures were driven by social and psychological effects of training [10]. Overall, gains in motor function measured with clinical outcome measures were possible in these three cases despite the addition of AS demands to training.

Task-oriented training is a key aspect of arm rehabilitation after stroke. The principles of motor skill learning have been suggested to be a key factor in the implementation of task-oriented training after stroke [8, 55], however, direct evidence on the effect of variations in practice conditions within the context of functional arm training on brain and behavior is limited. The ability to change the structure of task-oriented training to target a movement preparation process and its related neural network may provide a novel approach to using this behavioral toolbox during training. In the current case series, AS demands were added to each movement repetition in an effort to engage the AS process and the frontal and parietal brain regions that have been shown to support AS. We chose this movement preparation process because it is important in the performance of goal-direction actions [1], provided a clear, well-defined variation in practice condition and had a robust literature on its neural correlates [2–5, 26, 27], including PMd, a brain region thought to contribute to motor function after stroke [28–31]. However, different demands could be added to training to target other motor-cognitive processes and brain regions, such as self-directed choice to target supplementary motor area [56–58] or dual-task to target DLPFC [59–61]. Such targeted changes in practice conditions could allow clinicians to engage specific motor-cognitive processes and their neural correlates in addition to the motor execution system during task-oriented training.

This was a case series of three individuals with mild to moderate motor impairment in the chronic stage of stroke recovery. These three individuals tolerated the intervention and showed changes in AS performance and motor function. However, the feasibility and effectiveness of this approach beyond these three cases cannot be determined. Additionally, factors that may influence response to this training (i.e., lesion location or clinical presentation) are not possible in this case series. All three individuals received task-oriented arm training with AS demands. It is possible that the task-oriented training contributed to changes in AS behavioral performance and changes in brain activation during AS. Studies on the effect of task-oriented training on AS or motor planning are limited to a single small pilot study involving constraint-induced movement therapy [62]. It is not possible to fully disentangle these two features (task-oriented training, AS demands) in the current case series. AS demands were progressed to maintain some degree of challenge throughout training based on performance accuracy. While data driven, this approach may not have been optimal. Two participants (S2 and S3) achieved the highest level of cue difficulty midway through training suggesting additional difficulty levels (e.g., six cues) may be needed to maintain challenge for some individuals.

## 5. Conclusions

In conclusion, it was feasible to add AS demands to functional task-oriented arm training in three individuals with mild to moderate motor dysfunction poststroke. Improved AS behavioral performance was found after training that corresponded to a decrease in the neural resources required to complete an AS task. These gains in AS performance coincided with improvements in motor function suggesting that the addition of AS demands to training did not interfere with motor gains in these three individuals. The addition of AS demands to task-oriented arm training may provide an avenue to challenge motor preparation processes in addition to motor execution during functional training after stroke.

## Figures and Tables

**Table 1:** Participant demographics.

	S1	S2	S3
Age	54	67	63
Sex	Male	Male	Female
Arm dominance	Right	Right	Right
Side of hemiparesis	Right	Right	Right
Months poststroke	63	158	49
Comorbid conditions	HTN	HTN	HTN, DM
Lesion volume (cc)	177.8	116.6	3.3
Lesion location	L frontal, parietal, temporal, BG	L frontal, temporal, BG	L CR
CST FA (lesioned/nonlesioned)	0.70/0.70	0.56/0.61	0.64/0.067
NIH Stroke Scale	3	3	3
CES-D	4	3	3
Test for Upper Limb Apraxia (max 240)	229	238	240
MOCA (max 30)	18	19	21
MVPT-4 (max 100%)	3	27	18
Nottingham Sensory Scale (max 17)	5	17	17
UEFM Motor Score (max 66)	54	51	47

CST FA = Corticospinal Tract Fractional Anisotropy (range 0-1); CES-D = Center for Epidemiological Studies Depression Scale; MOCA = Montreal Cognitive Assessment; MVPT = Motor-Free Visual Perceptual Test, scores represent age-normed percentile scores; UEFM = Upper Extremity Fugl-Meyer; HTN = Hypertension; DM = Diabetes Mellitus; L = left; BG = Basal ganglia; CR = Corona radiata.

**Table 2:** Example of action selection cues during training.

	Bimanual Symmetrical task	Unimanual Fine motor task	Unimanual Object manipulation
Task	Pick up a large stock pot, move, and release	Pick up a dime and insert into bank	Spoon beans from one bowl to another bowl
Start position	1 pot at midline	2 piles of dimes Bank placed 10 cm in front at midline	One bowl with beans at midline Two bowls for receiving beans
Select cue directs	Direction of movement	Pile for picking up dime	Bowl to deposit beans
Select cue orientation:			
Right–left	45° to the right 45° to the left	10 cm to right of midline 10 cm to left of midline	45° to the right 45° to the left
Close – Far	10 cm forward 20 cm forward	5 cm and 45° to right 10 cm and 45° to right	10 cm and 45° to left 20 cm and 45° to left
High – Low	Level on training table Shelf 20 cm above training table	Level on training table Shelf 20 cm above training table	Level on training table Shelf 20 cm above training table

**Table 3:** Changes in clinical measures with training.

	S1	S2	S3
ARAT (max 57)			
Baseline 1	54	55	42
Baseline 2	55	55	42
Post-TX	54	55	55^∗^
Follow-up	54	56	53^∗^
BBT (# blocks)			
Baseline 1	37	50	22
Baseline 2	40	50	32
Post-TX	42	55	35
Follow-up	40	50	36
SIS Hand Domain (max 100)			
Baseline 1	50	60	45
Baseline 2	50	40	60
Post-TX	70^∗^	80^∗^	95^∗^
Follow-up	75^∗^	75^∗^	95^∗^
UEFM Motor Score (max 66)			
Baseline	54	51	47
Post-TX	54	55	48
Follow-up	54	48	53
Nine-Hole Peg Test (sec)			
Baseline	47.06	36.37	56.34
Post-TX	40.03	23.0	51.84
Follow-up	43.5	35.19	46.1
CAHM (max 100)			
Baseline	59.5	80.0	71.5
Post-TX	79.5^∗^	79.5	93.5^∗^
Follow-up	81.5^∗^	86.0	96.0^∗^

^∗^Change greater than minimally clinically important difference for that measure; ARAT = Action Research Arm Test; BBT = Box & Blocks Test; SIS = Stroke Impact Scale; UEFM = Upper Extremity Fugl-Meyer; CAHM = Confidence in Arm and Hand Movements.

## Data Availability

The data used to support the findings of this study are available from the corresponding author upon request.

## Supplementary Materials

Supplementary Materials The following Supplementary Materials are available for this paper: joystick and brain imaging data analysis methods, supplemental table listing location of activation peaks, and supplemental figures showing stroke lesion location, behavioral performance, and imaging data.
