# Supplementary material for: Targeted Engagement of the Action Selection Network during Task-Oriented Arm Training after Stroke
Source: Neural Plast. 2020 Sep 23;2020:8814158. doi: 10.1155/2020/8814158 (PMC7530513; doi:10.1155/2020/8814158)
Supplement: Supplementary Materials — The following Supplementary Materials are available for this paper: joystick and brain imaging data analysis methods, supplemental table listing location of activation peaks, and supplemental figures showing stroke lesion location, behavioral performance, and imaging data. [file 8814158.f1.pdf]

Supplemental Methods and Results

Targeted engagement of the action selection network during task-oriented arm training after stroke: a case series

Jill Campbell Stewart[1], Kaci Handlery[1], Jessica Baird[2], Erika Blanck[3], Geetanjali Pathak[2], Stacy L. Fritz[1]

[1]Physical Therapy Program, Department of Exercise Science, University of South Carolina
[2]Department of Exercise Science, University of South Carolina
[3]Department of Cell Biology and Anatomy, School of Medicine, University of South Carolina

Corresponding Author: Jill Campbell Stewart, PT, PhD
University of South Carolina
921 Assembly Street, Room 301D
Columbia, SC 29208
PHONE: (803) 777-6583
FAX: (803) 777-0558
EMAIL: jcstewar@mailbox.sc.edu

Supplemental Methods

Brain-Behavior Probe

Joystick Data Analysis

Data from the joystick during practice were used to determine task accuracy and reaction time using a custom script in Matlab (Matworks, Inc., Natick, MA). Position data (x,y) were recorded throughout each trial (60 Hz) and used to derive movement velocity [1]. Reaction time (RT), the primary behavioral outcome measure, was the time between cue presentation and movement onset. Movement onset was determined by searching backward in time from initial peak velocity until velocity dropped below 5°/sec for two consecutive samples or the change in velocity dropped below 1°/sec for two consecutive samples, whichever was identified first. Movement offset was determined by searching forward in time from peak velocity until velocity dropped below a minimum velocity (10°/sec if peak velocity was <30; 40°/sec if peak velocity was >100; 25°/sec if peak velocity was between 30 and 100) and either changed directions or the change in velocity was below 5°/sec. Accuracy (correct movement direction), reaction time (movement onset time-cue onset time; accurate trials only), and movement time were extracted for each trial. Select RT was normalized to Execute RT to determine RT cost (Select RT–Execute RT), a measure of the relative increase in planning time for the Select condition for each participant.

Diffusion Weighted Imaging Analysis

Corticospinal tract integrity was quantified using the diffusion weighted images. Analysis was completed in FSL (FMRIB Center, Oxford, UK) using the FDT toolbox [2].

Diffusion images were corrected for eddy currents and head motion followed by removal of the skull and dura [3]. A voxelwise map of fractional anisotropy (FA) was then created. FA is a measure of the structural integrity of white matter with values ranging between 0 and 1 with higher values indicating greater structural integrity [4]. To determine corticospinal tract integrity, a region of interest (ROI) mask as manually drawn on the three contiguous axial slices that showed the largest cross-sectional area of the cerebral peduncle [5,6]. Mean FA was extracted from each ROI using a threshold of FA>0.2.

Supplemental Table 1. Action Selection Cue Progression During Training

Action Selection Cue Difficulty Level	Description	Criteria to Move to Next Level
1	2 Cue Set	>85% accuracy for 1 day
2	New 2 Cue Set Each Day	>95% accuracy for 2 days
3	New 2 Cue Set Each Task	>95% accuracy for 2 days
4	New 2 Cue Set Each 10 Trial Block	> 95% accuracy for 2 days
**5**	**4 Cue Set – Start Point**	**>85% accuracy for 1 day**
6	New 4 Cue Set Each Day	>95% accuracy for 2 days
7	New 4 Cue Set Each Task	>95% accuracy for 2 days
8	New 4 Cue Set Each 10 Trial Block	>95% accuracy for 2 days

All participants began at level 5 on the first day of training and progressed as indicated. Individuals who were <70% accurate for movement selection on Day 1 would move to level 1 (2 cues instead of 4 cues) and progress as indicated.

Supplemental Table 2. Peak Activation Locations

	S1			S2			S3		
	X	Y	Z	X	Y	Z	X	Y	Z
Left PMd	-24	-12	60	-26	-14	50	-26	-14	68
Left DLPFC	-34	52	26	-32	26	30	-38	48	12
Left Parietal	-26	-66	48	-24	-56	42	-32	-60	40
Right PMd	22	0	52	26	-8	48	28	12	60
Right DLPFC	40	44	28	36	36	28	32	46	12
Right Parietal	36	-58	48	16	-60	54	42	-46	52

MNI coordinates for peak of activation during Select performance with the contralesional, right hand. All peaks were significant at $p<0.05$ with a family-wise error correction. PMd=Dorsal premotor cortex; DLPFC=Dorsolateral prefrontal cortex

Supplemental Figure 1. Stroke Lesions

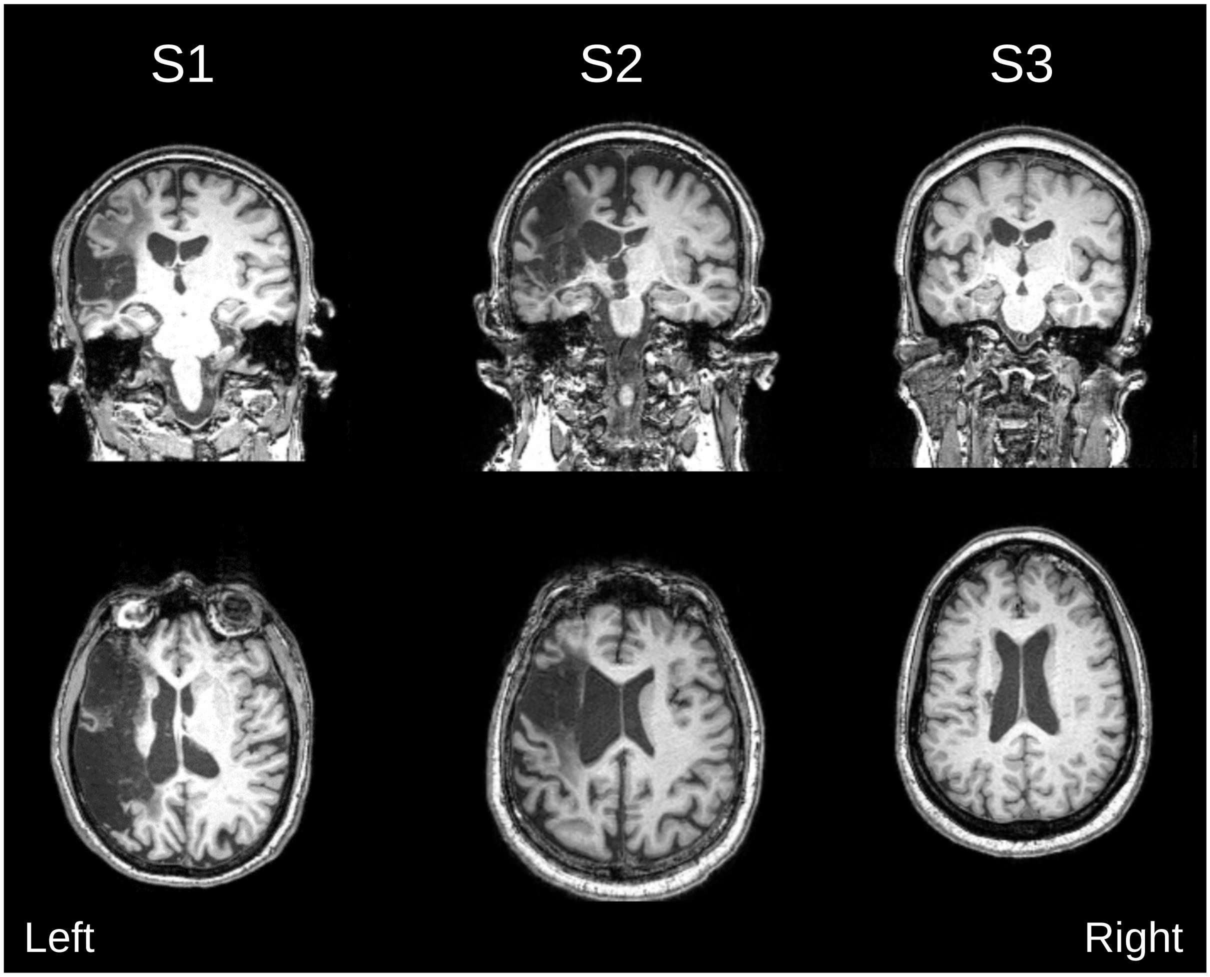

Supplemental Figure 1. Stroke lesions shown in T1 structural images on coronal slices (top row) and axial slices (bottom row). All lesions were in the left hemisphere.

Supplemental Figure 2. Behavioral Performance on Action Selection Task

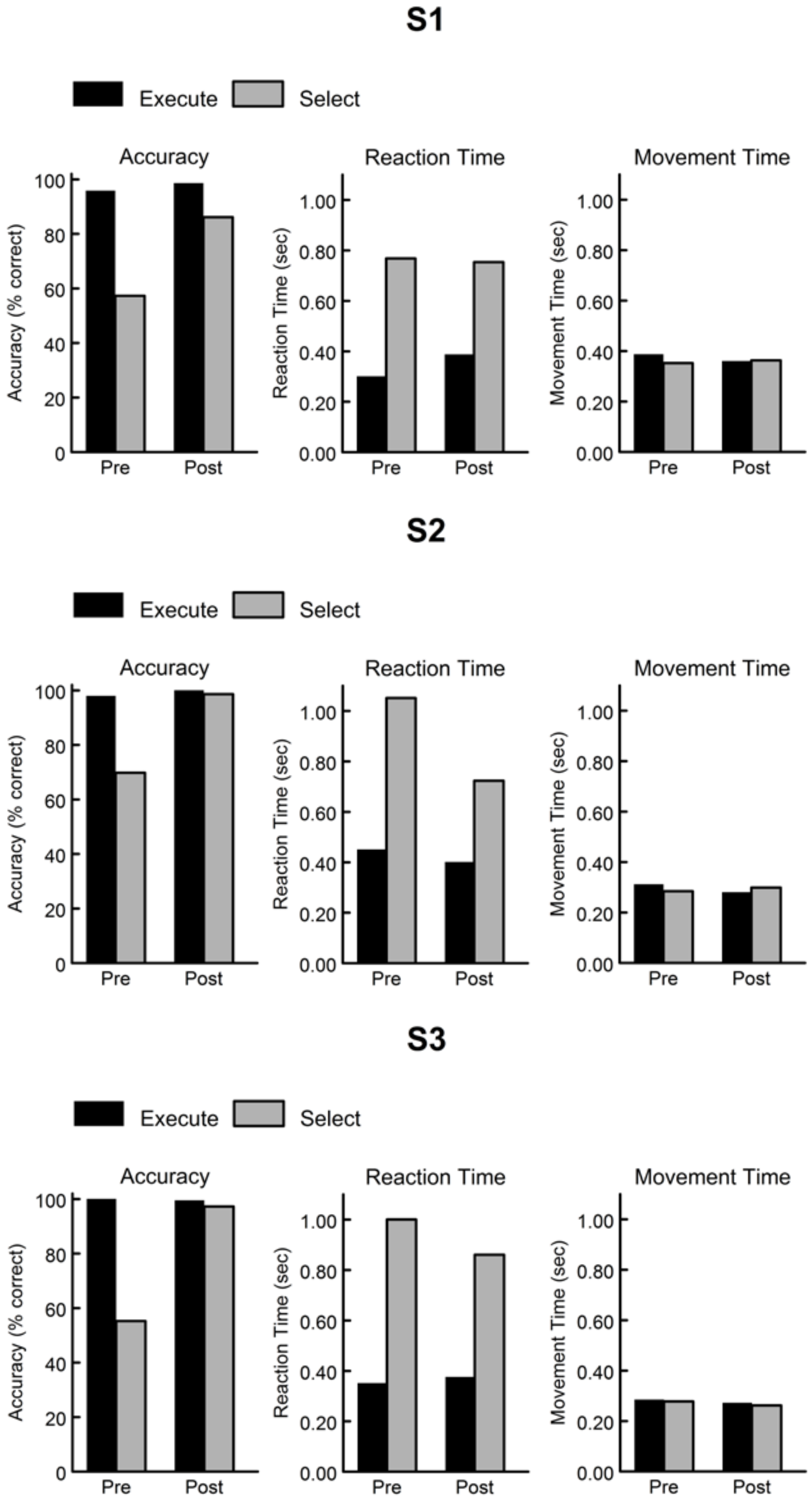

Supplemental Figure 2. Behavioral performance on action selection task before (Pre) and after (Post) motor training. Data represents mean performance for all blocks on each day.

Supplemental Figure 3. Brain Activation by Condition and Day

S1

A.

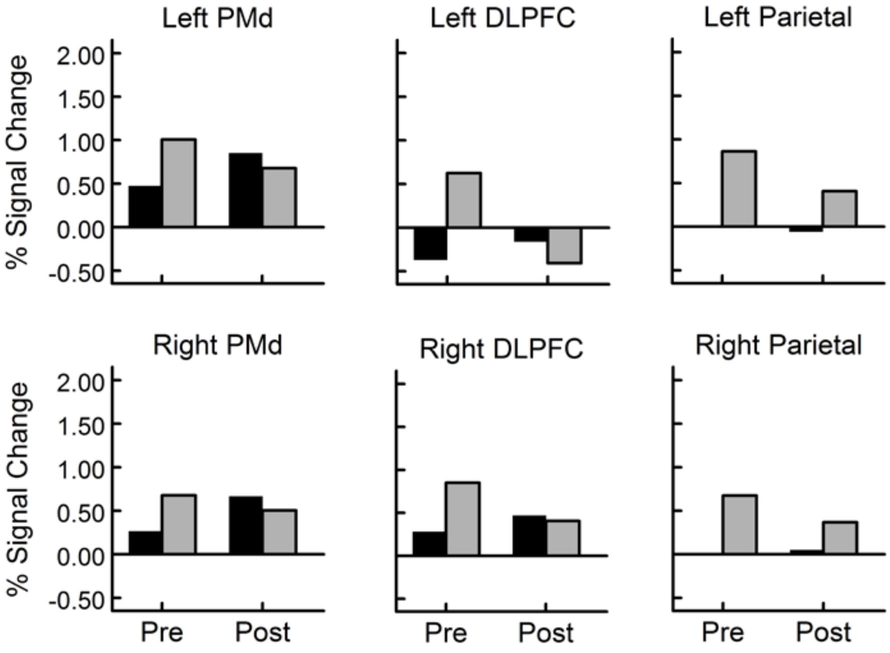

S2

B.

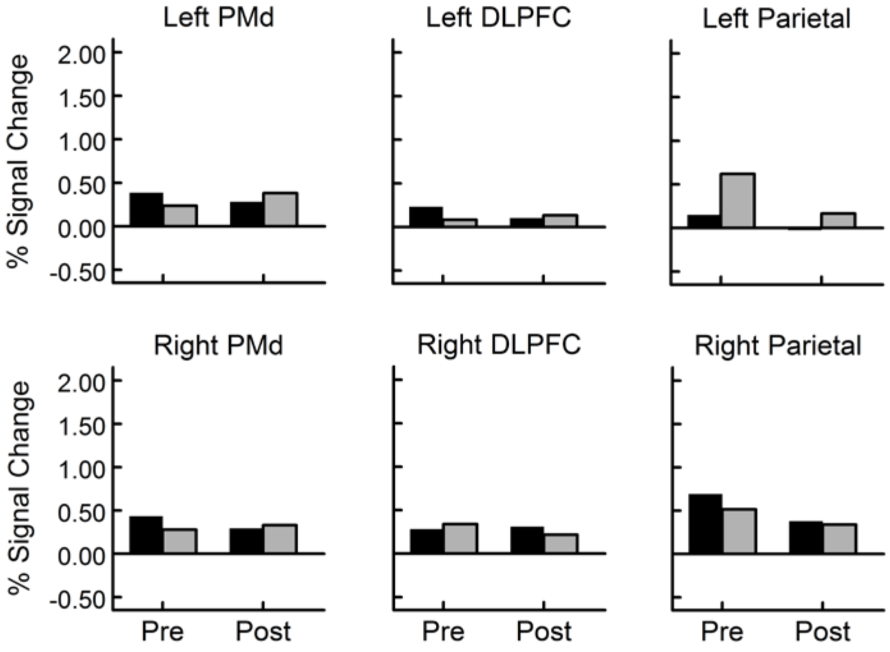

S3

C.

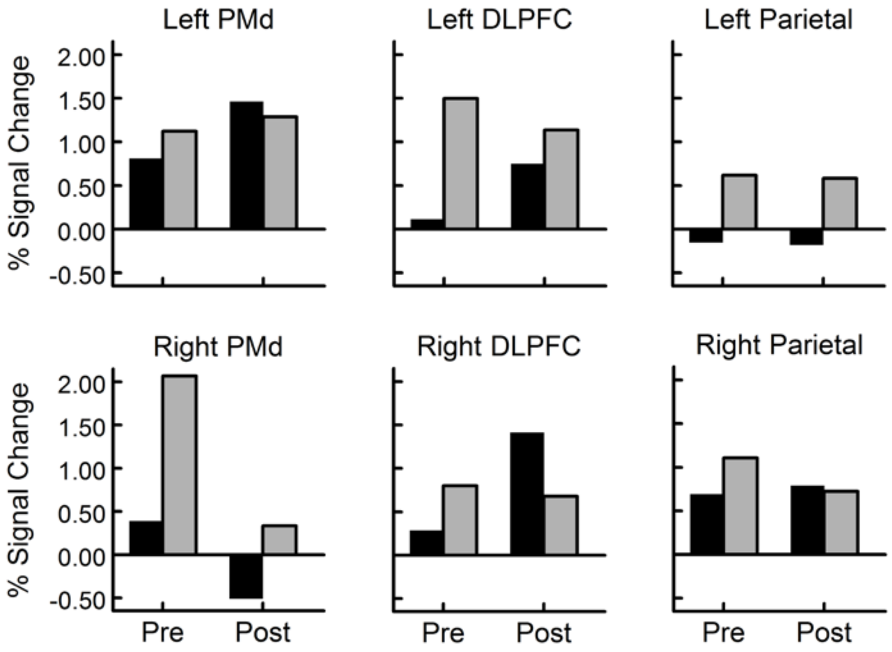

Supplemental Figure 3. Brain activation (% Signal Change during movement compared to rest) in regions of interest during Execute (black) and Select (grey) before (Pre) and after (Post) motor training for S1 (A), S2 (B), and S3 (C). PMd=dorsal premotor cortex; DLPFC=dorsolateral prefrontal cortex.

References

1. Winter D. Biomechanics and motor control of human movement. 3 ed. Hoboken: John Wiley & Sons, Inc; 2005.
2. Behrens TE, Woolrich MW, Jenkinson M, et al. Characterization and propagation of uncertainty in diffusion-weighted MR imaging. Magn Reson Med. 2003 Nov;50(5):1077-88.
3. Smith SM. Fast robust automated brain extraction. Hum Brain Mapp. 2002 Nov;17(3):143-55.
4. Mori S, Zhang J. Principles of diffusion tensor imaging and its applications to basic neuroscience research. Neuron. 2006 Sep 7;51(5):527-39.
5. Mark VW, Taub E, Perkins C, et al. Poststroke cerebral peduncular atrophy correlates with a measure of corticospinal tract injury in the cerebral hemisphere. AJNR Am J Neuroradiol. 2008 Feb;29(2):354-8.
6. Burke E, Dodakian L, See J, et al. A multimodal approach to understanding motor impairment and disability after stroke. J Neurol. 2014 Jun;261(6):1178-86.
